# A comparative assessment of conventional and molecular methods, including MinION nanopore sequencing, for surveying water quality

**DOI:** 10.1038/s41598-019-51997-x

**Published:** 2019-10-31

**Authors:** Kishor Acharya, Santosh Khanal, Kalyan Pantha, Niroj Amatya, Russell J. Davenport, David Werner

**Affiliations:** 1 School of Engineering, Newcastle University, Newcastle upon Tyne, NE1 7RU United Kingdom; 2 Department of Pharmacology, School of Medicine, University of Colorado, Aurora, Colorado, 80045 USA; 3 Group for Rural Infrastructure Development, Wise use House, Jwagal, Lalitpur, Nepal; 4 Faculty of Chemistry, University Duisburg-Essen, Universitätsstr. 5, D-45141 Essen, Germany; 5 Department of Medical Microbiology, Nobel College, Pokhara University, Kathmandu, Nepal

**Keywords:** Environmental microbiology, Metagenomics, Environmental sciences, Hydrology

## Abstract

Nucleic acid based techniques, such as quantitative PCR (qPCR) and next generation sequencing (NGS), provide new insights into microbial water quality, but considerable uncertainty remains around their correct interpretation. We demonstrate, for different water sources in informal settlements in the Kathmandu Valley, Nepal, significant Spearman rank correlations between conventional and molecular microbiology methods that indicate faecal contamination. At family and genera level, 16S rRNA amplicon sequencing results obtained with the low-cost, portable next generation sequencer MinION from Oxford Nanopore Technologies had significant Spearman rank correlations with Illumina MiSeq sequencing results. However, method validation by amplicon sequencing of a MOCK microbial community revealed the need to ascertain MinION sequencing results for putative pathogens at species level with complementary qPCR assays. *Vibrio cholerae* hazards were poorly associated with plate count faecal coliforms, but flagged up by the MinION screening method, and confirmed by a qPCR assay. Plate counting methods remain important to assess viability of faecal coliforms in disinfected water sources. We outline a systematic approach for data collection and interpretation of such complementary results. In the Kathmandu Valley, there is high variability of water quality from different sources, including for treated water samples, illustrating the importance of disinfection at the point of use.

## Introduction

An estimated 1.8 billion people are still exposed to drinking water sources contaminated with faecal matter^1^. In Nepal waterborne diseases account for 15% of all illness and 8% of total deaths^2^. With waterborne diseases accounting for 38% of deaths in children under the age of five^3^, water quality in Nepal is a major public health concern^2,4–6^. According to the Central Bureau of Statistics, Government of Nepal, one out of five families in the Kathmandu Valley does not have access to municipal drinking water, and in most areas availability is <4–7 h/week^3,7^. Many people depend on alternative sources such as groundwater from boreholes and wells, stone spouts, bottled drinking water or water supplied by delivery truck^2,6,8^. The quality of these water sources is not routinely monitored.

The current standard method for microbial examination of both drinking and bathing water requires the isolation and enumeration of organisms that indicate the presence of faecal contamination (i.e. faecal indicator organisms, FIO)^9^. *Escherichia coli* are World Health Organization (WHO) recommended faecal indicator bacteria for drinking water^10^, while *Enterococci* are indicator bacteria for bathing water^11^. FIO are used because there is good epidemiological evidence that they correlate with disease outcomes^12,13^. However, FIO are a poor proxy for organisms with quite different physiologies (e.g. viruses and protozoa) and it is difficult to distinguish FIO from human or animal sources^14^. The routine isolation of all pathogens is impractical^15^, as each requires a unique microbiological isolation technique^16,17^. In addition, culture dependent approaches may require long incubation periods, and there is a demand for more rapid and comprehensive screening methods to detect FIO and/or their markers^14^ and putative pathogens in water samples^15,18–21^. Culture independent methods such as quantitative real-time PCR (qPCR)^14^, next generation sequencing (NGS)^22^, DNA hybridisation platforms and immunoassays^23^ allow direct measurement of cellular properties that may identify pathogens (e.g., DNA, RNA, cellular proteins) without incubation. These methods may reduce the detection and quantification time to a few hours^19,20,24^. NGS methods potentially allow simultaneous detection of gene fragments from different types of faecal indicators and putative pathogens, such as thermo-tolerant coliforms, faecal coliforms, *Vibrio cholerae*, *Streptococci*, *Bacteroides*, etc., especially through 16S rRNA gene amplicon sequencing (from here on referred to as amplicon sequencing). However, NGS techniques are typically more expensive and require more sophisticated equipment and reagents than culturing methods^18,24–26^. Also, NGS techniques may have limited taxonomic resolution; due to an inability to capture the near-complete genomes of rare taxa in shot-gun sequencing or an inability to reliably classify taxa down to the species level with amplicon sequencing due to the relatively short fragment sizes that can be sequenced on some NGS platforms, or the presence of highly conserved 16S rRNA genes in some families and genera^22,27,28^. This compromises their ability to reliably detect waterborne hazards. To circumvent some of these limitations, different approaches can be combined. For instance, *Cui et al*. combined Illumina-based amplicon sequencing and qPCR to evaluate the pathogen diversity in urban recreational water^29^. *Ahmed et al*. used host associated molecular markers with qPCR and Illumina-based amplicon sequencing to identify faecal pollution sources in environmental waters in Brisbane, Australia^30^. An exciting development for NGS is the MinION, a low cost, memory-stick sized real time sequencer from Oxford Nanopore Technologies Ltd. Its portability opens up the possibilities for sequencing to be done in the field. Recently, *Hu et al*. compared culturing, MinION shotgun sequencing and Illumina MiSeq amplicon sequencing methods to trace faecal contamination from wastewater in urban stormwater systems^24^. They demonstrated high correlations between *E. coli* culturing counts, the relative abundance of human gut microbiome related amplicon sequences, and the frequencies of human gut microbiome genes from shotgun sequencing data. However, they did not assess their method validity using samples of known composition. When applied to drinking water quality monitoring, qPCR and NGS data were not always found to be well-correlated with culture based methods^18,31^. Reliable identification of putative pathogens at species level remains a challenge, especially for water samples containing low amounts of DNA.

To address these uncertainties, we first evaluated various molecular microbiology methods (qPCR, Illumina and MinION NGS) using samples of known composition (i.e. MOCK communities containing DNA of FIO and putative pathogens). Since our samples of interest would include groundwater and treated drinking water samples with low amounts of DNA, we focused on the evaluation of qPCR and NGS 16S rRNA amplicon sequencing methods. We combined these molecular with conventional methods to assess water quality for different types of household water sources in the Kathmandu Valley, Nepal. To our knowledge, this is the first study using amplicon sequencing with the MinION for microbial water quality analysis, and comparing its results with those obtained with other methods and known sample compositions. Based on the results, we propose a tool-box approach to make the most of complementary technologies for water quality monitoring in areas with a significant waterborne disease burden.

## Results

### MOCK microbial community analysis

**Figure 1:**
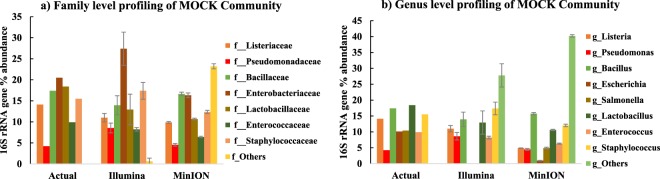
Results of the experimentally determined composition of the MOCK Community (MC) at (**a**) family and (**b**) genus level with MinION and Illumina NGS techniques. The results are based on 16S rRNA amplicon sequencing. Experimentally determined 16S rRNA gene percentage abundance are compared against the actual composition of the MC as reported by the supplier (i.e. Zymo Research). Data points are an average of duplicate samples for Illumina and triplicate samples for MinION sequencing. Error bars indicate the standard deviation.

**Figure 2:**
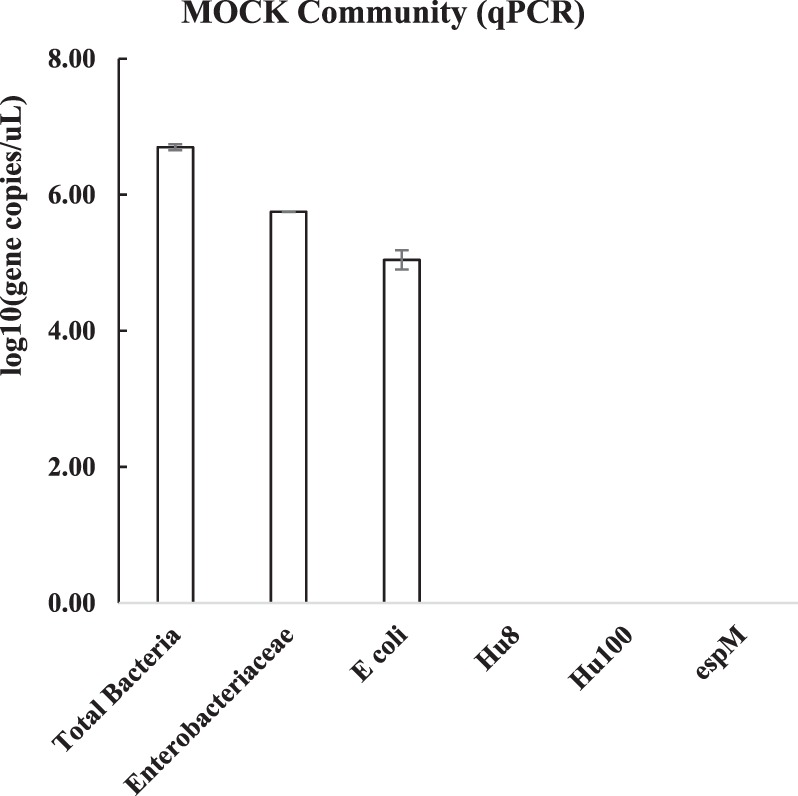
Molecular microbiology analysis of the MOCK community. Data points are an average of technical triplicate samples and error bars indicate the standard deviation. Genes associated with human *E. coli* and *Vibrio cholerae* were not detected.

For method validation, we used a MOCK community consisting of genomic DNA from eight bacterial and two fungal species. The MOCK community included Gram-negative and Gram-positive faecal indicator bacteria (*Escherichia coli*, and *Enterococcus faecalis*, respectively) and other putative pathogens (*Salmonella enterica* and *Pseudomonas aeruginosa*) (Table S1 in supporting information and Fig. 1). The percentage abundance of 16S rRNA genes from each species provided by the supplier of the MOCK community (i.e. Zymo Research) is considered as the true or actual abundance in this study. The MOCK community contained an equal ratio of genomic DNA for each species. However, different 16S rRNA gene copy numbers for each species leads to a slightly uneven relative abundance for this gene (Table S1). Figure 1 and Table S2 in supporting information compare the actual composition of the MOCK community with that measured by two different NGS methods at family, genus and species level. We sequenced 16S rRNA gene amplicons from the MOCK community using two NGS methods, the portable, memory-stick sized MinION of Oxford Nanopore Technologies, and the MiSeq platform from Illumina, which is currently the most commonly used for microbial community analyses^22,25,32^. Illumina sequencing data showed better taxonomic resolution as compared to MinION data at the family level, since 99% of Illumina reads were correctly classified as those bacterial families present in the MOCK community compared to 76.79% for MinION reads. However, at genus and species level, MinION sequencing data showed better taxonomic resolution as compared to Illumina data (Fig. 1b for genus level and Table S2 for species level). In this study, the presence of the genera *Escherichia* and *Salmonella* in the MOCK community was not identified from the Illumina data, and none of the sequencing reads from Illumina were classified to species level. In contrast, almost 59.41% of the MinION sequencing data were classified to species level. However, at species level, 64.28% of MinION reads were matched to species that were not present in the MOCK community (Table S2). In particular, reads falsely classified as *Escherichia fergusonii* had a higher relative abundance than *Escherichia coli*, 0.65% versus 0.17%, respectively, which compares to their true relative abundances of 0% and 10.1%, respectively in the MOCK community (Tables S1 and S2). In comparison with *E. coli*, MinION sequencing more successfully identified the other faecal pollution indicator, *Enterococcus faecalis*, in the MOCK community. *Enterococcus faecalis* was the most frequently detected *Enterococcus* species, with a measured 16S rRNA read abundance of 5.39% compared to the actual or true 16S rRNA gene abundance of 9.9% (Tables S1 and S2). For the other putative pathogens, *Salmonella enterica* was the most frequently detected *Salmonella* species with a measured 16S rRNA reads abundance of 4.89% compared to a true 16S rRNA gene abundance of 10.4%, and *Pseudomonas aeruginosa* was the most frequently detected *Pseudomonas* species with 3.66% measured abundance compared to a true abundance of 4.2% (Tables S1 and S2). Cross-validation of sequencing results, such as targeting and quantifying marker genes with PCR primers can help in interpreting and affirming sequencing outcomes^30,33^. When using total 16S rRNA, total coliforms, total *E. coli*, human *E. coli* and *Vibrio cholerae* marker gene primers to analyse the MOCK community (Fig. 2), we only identified genes associated with *Enterobacteriaceae* (i.e. total coliform) and *E. coli* (i.e. rodA), but not *Vibrio cholerae* and human *E. coli* genes. These results are consistent with the true or actual composition of the MOCK community, since the *E. coli* DNA in the MOCK community was not from a strain associated with the human gut microbiome.

### Method comparison for the characterisation of microbial water from different sources in the Kathmandu Valley, Nepal

**Figure 3:**
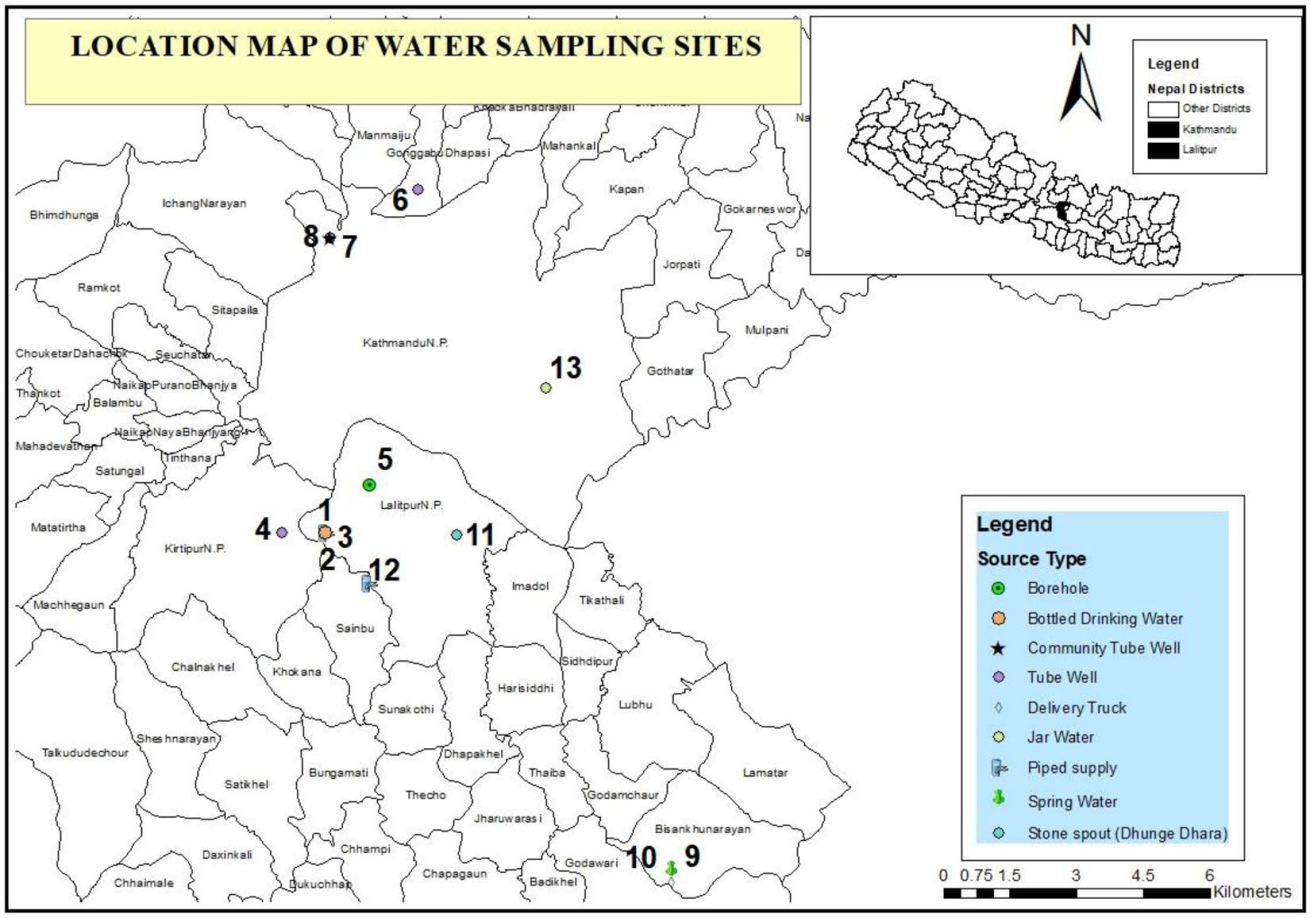
Map showing the location of the 13 sampling sites in the Kathmandu Valley and types of water sources sampled.

**Figure 4:**
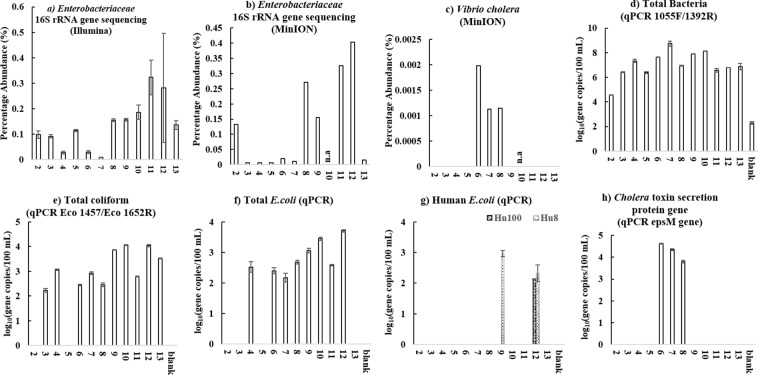
Molecular microbiology analysis of water samples from different water sources in the Kathmandu Valley. Numbers on the x-axis indicate the ID of the different sampling sites as described in Table 1. Data points for qPCR results are an average of technical triplicate samples and error bars indicate the standard deviation. Data points for Illumina NGS sequencing are an average of duplicate samples and error bars indicate the standard deviation. Library preparation for MinION NGS was unsuccessful for the sample from location 10 and thus data are not available (n.a), while the DNA extraction yield for the blank was too low for NGS, and for the sample from location 1, the yield was too low for all molecular microbiology methods.

**Figure 5:**
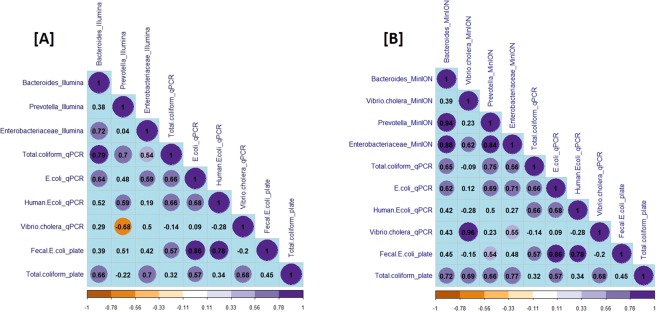
Result of correlation analysis between different microbial water quality indicators determined with different methods: plate count, qPCR and NGS (**A**: Illumina and **B**: MinION). The colour intensities of circles are proportional to the correlation coefficients (Spearman rank correlation, p < 0.05, n = 12), and the circled numbers represents statistically significant coefficients.

NGS (Illumina and MinION), qPCR and plate count methods were combined to investigate microbial water quality for thirteen water sources in the Kathmandu Valley, Nepal (Fig. 3 and Table 1). The data is shown in Fig. 4, and Tables S3–S7 in supporting information. First, the agreement between the two NGS methods was evaluated for several relevant groups of bacteria that include faecal indicators (*Bacteroides, Prevotella*, *Enterobacteriaceae)*, and other groups containing putative pathogens (i.e. *Vibrio, Pseudomonas, Legionella, Clostridium* and *Streptococcus*), distinguished at family (*Enterobacteriaceae)* or genus level (*all others*). A significant rank correlation was observed (Spearman rank correlation coefficient, p < 0.05) between the relative abundances of *Bacteroides, Prevotella*, *Enterobacteriaceae* and all other putative pathogenic genera determined by MinION and Illumina NGS (Table S6 in supporting information). The extent of rank correlation between the NGS and other approaches for microbial water quality assessment is illustrated in Fig. 5 separately for NGS data from Illumina [A] and the MinION [B]. To enable this correlation analysis between NGS data (which is relative abundance) and qPCR and plate count methods (which provide absolute abundance), NGS results were first converted to estimated absolute abundances using the 16S rRNA gene copies number determined by qPCR for each sample (Fig. 4d)^21^. When looking at the agreement between NGS and qPCR methods, *Enterobacteriaceae* Illumina data (Fig. 5A) had a significant correlation with the total coliform qPCR (Spearman rank correlation coefficient 0.54, p < 0.05), total *E. coli* qPCR (Spearman rank correlation coefficient 0.59, p < 0.05), but not with human *E. coli* qPCR (Spearman rank correlation coefficient 0.19, p > 0.05) gene copy numbers. Likewise, *Enterobacteriaceae* MinION data (Fig. 5B) had a significant correlation with total coliform qPCR (Spearman rank correlation coefficient 0.56, p < 0.05) and total *E. coli* qPCR (Spearman rank correlation coefficient 0.71, p < 0.05), but not with human *E. coli* qPCR (Spearman rank correlation coefficient 0.27, p > 0.05) gene copy numbers. As expected from the MOCK community results, only the NGS results from the MinION contained several reads aligned with putative pathogens at species level (Table S7 in supporting information). For the water samples from the Kathmandu Valley, *Vibrio cholerae* MinION data (Fig. 4c) were well aligned with qPCR results (Fig. 4h and Table S7), which were targeting copies of the gene encoding for the cholera toxin secretion protein epsM as an indicator for the abundance of pathogenic *Vibrio cholerae* strains. These genes were generally detected only in the samples from tube wells (location 6, 7 and 8), which were also the only samples for which NGS with the MinION matched some reads to *Vibrio cholerae* at species level. With regards to the agreement between molecular and plate count methods, all MinION NGS data had a significant and positive correlation with the total coliform plate count data (Spearman rank correlation coefficients > 0.66, p < 0.05). Positive correlations were also observed between faecal *E. coli* plate count and total coliform qPCR, total *E. coli* qPCR, and human *E. coli* qPCR data, respectively (Spearman rank correlation coefficients > 0.57, p < 0.05). The correlation between faecal *E. coli* plate count and total *E. coli* qPCR data was particularly strong (Spearman rank correlation coefficient 0.86, p < 0.05). It is noteworthy that *Vibrio cholerae* MinION and *Vibrio cholerae* qPCR data were negatively correlated with the faecal *E. coli* plate count (Fig. 5B). Consequently, the *Vibrio cholerae* hazard is not readily detected with the classic fecal pollution indicator method. While the overall agreement between the traditional (i.e. plate count) and molecular microbial water quality assessment methods was thus generally good, there were noteworthy discrepancies for disinfected water samples. Water sampled from location 2, 3 and 13 were disinfected waters (2 was disinfected by the household at the point of use while 3 and 13 were commercially sold bottled water), and showed no evidence of thermo-tolerant coliform bacteria by the plate count method (Table S3 in supporting information). However, these groups of bacteria were detected in samples 3 and 13 in significant numbers when analysed with qPCR (Fig. 4e).

**Table 1:** Water sampling locations, water types, and additional details about the water sources.

Sample Site ID	Location	Source Type	Latitude	Longitude	Depth to water (m)	Treatment	Additional Protection	Sample collected date	Water Usage	Molecular analysis
1	Dhobighat, Lalitpur	Piped supply	27.673904	85.29678	Ground level	Treated	None	30.10.2018	Washing, Bathing, Gardening	No
2	Dhobighat, Lalitpur	Piped supply	27.673904	85.29678	Ground level	RO/UV treated by houseowner	None	30.10.2018	Drinking	Yes
3	Dhobighat, Lalitpur	Bottled Drinking Water	27.674173	85.297901	Ground level	RO/UV treated	Sealed bottle	30.10.2018	Drinking	Yes
4	Dhobighat, Lalitpur	Tube Well	27.673718	85.294829	10	Untreated	None	30.10.2018	Washing, Bathing, Gardening	Yes
5	Sanepa Height, Lalitpur	Borehole	27.683564	85.306661	91.5	Untreated	None	30.10.2018	Drinking	Yes
6	Basundhara, Kathmandu	Tube Well	27.74304	85.321844	9	Untreated	None	31.10.2018	Washing, Bathing, Gardening	Yes
7	Banasthali, Kathmandu	Tube Well	27.728475	85.295282	9	Untreated	None	31.10.2018	Washing, Bathing, Gardening	Yes
8	Banasthali, Kathmandu	Community Tube Well	27.728501	85.295739	12	Untreated	None	31.10.2018	Washing, Bathing, Gardening, Drinking	Yes
9	Godabari, Lalitpur	Spring Water	27.597466	85.384897	Ground level	Untreated	Mesh Covered	31.10.2018	Washing, Bathing, Gardening, Drinking	Yes
10	Godabari, Lalitpur	Delivery Truck	27.597315	85.384829	Ground level	Untreated	Locked Container	31.10.2018	Washing, Bathing, Gardening, Drinking	Yes
11	Mangalbazar, Lalitpur	Stone spout (Dhunge Dhara)	27.673638	85.32535	Ground level	Untreated	None	31.10.2018	Washing, Bathing, Gardening, Drinking	Yes
12	Kusunti, Lalitpur	Piped supply	27.664901	85.308355	Ground level	Treated	None	31.10.2018	Washing, Bathing, Gardening, Drinking	Yes
13	Sinamangal, Kathmandu	Jar Water	27.698192	85.346953	Ground level	Treated	Sealed Jar	31.10.2018	Drinking	Yes

### Chemical water quality assessment outcomes for different water sources in the Kathmandu Valley

Exceedances of the Nepalese guidance values were observed for the following chemical water quality parameters and locations: Nitrate in water from the stone spout (location 11) and a tube well (location 8), ammonia in one tube well (location 7), iron in location 5 (deep borehole) and location 7 (tube well), manganese in location 5 (deep borehole) and location 7 (tube well), and aluminum in location 4 (tube well). More details are provided in Tables S8–10 in supporting information.

## Discussion

**Figure 6:**
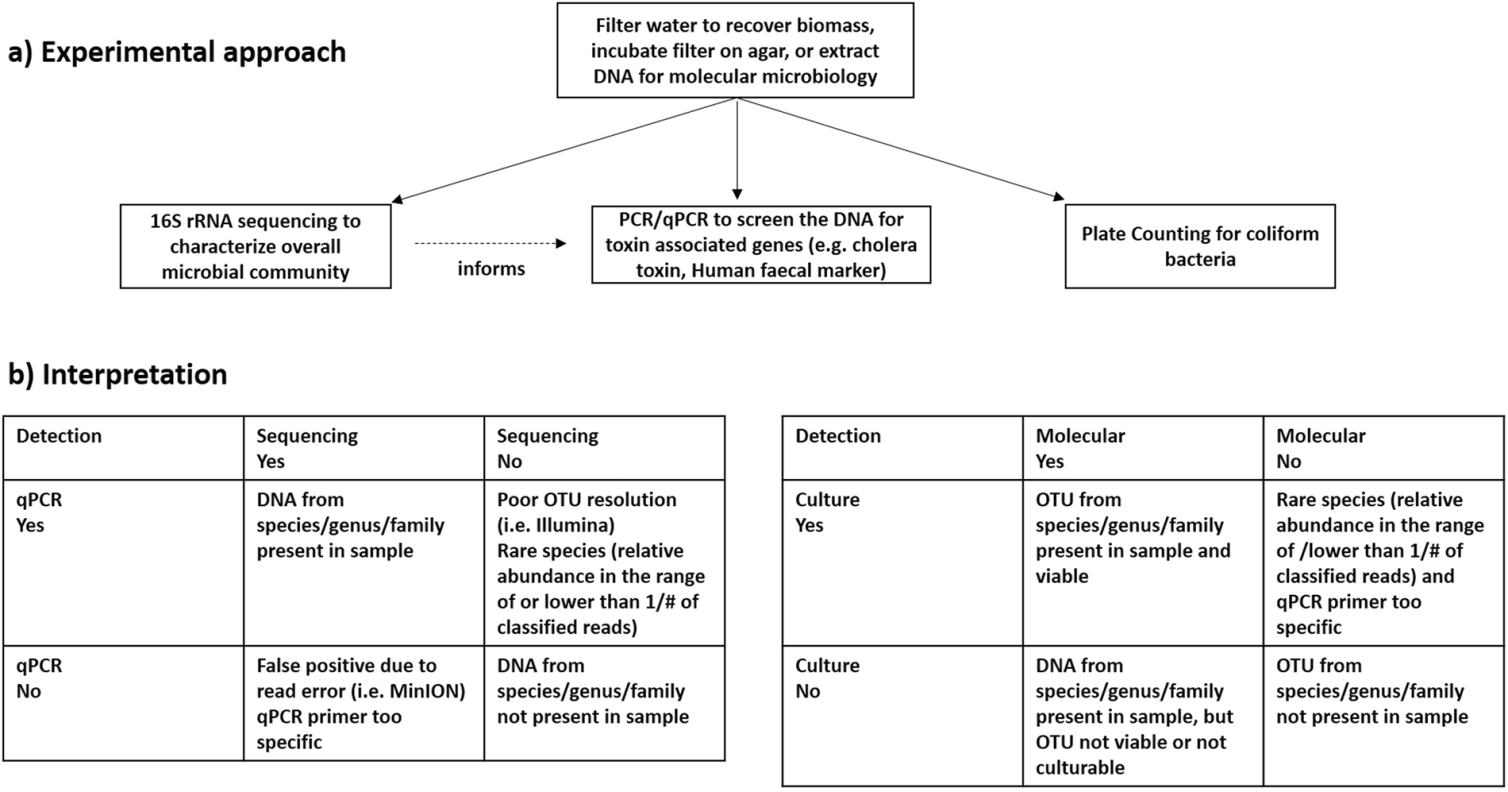
Outline of the experimental approach and methods (both traditional and DNA based) used for microbial water quality analysis, and the interpretation of water quality data obtained with those methods. Sample contamination due to non-sterile working practice may also explain discrepancies between different methodological approaches.

Analysis of a MOCK community revealed that Illumina sequencing data showed better taxonomic resolution as compared to MinION data at the family level, while MinION sequencing data showed better taxonomic resolution at genus and species level. The amplicon size of the targeted 16S rRNA gene sequences was shorter with Illumina than those targeted using the MinION, and short amplicon size is known to compromise the achieved taxonomic resolution when classifying sequences to taxa^22,27^. In this study, the presence of the genera *Escherichia* and *Salmonella* in the MOCK community was not identified from the Illumina data, and none of the sequencing reads from Illumina were classified to species level. In the context of water quality monitoring, this may then result in false negative outcomes at genus and species level for these putative pathogens. MinION sequencing has the advantage of a longer amplicon size, but the disadvantage of a reported higher error rate/lower accuracy than Illumina sequencing^27^. These MinION sequencing errors caused significant underreporting of the relative abundance of actual MOCK community members such as *Escherichia coli*. At the same time, assignment of partially erroneous reads to species by the MinION 16S bioinformatics workflow resulted in mismatched identities and taxonomic classification at species level, i.e. false positive results. While MinION sequencing detected all the species present in the MOCK community, these species were not always those that were most frequently detected within a certain genus. In particular, reads falsely classified as *Escherichia fergusonii* had a higher relative abundance than *Escherichia coli*. Phylogenetic analyses revealed that the 16S rRNA sequences for different strains of *E. coil*, *E. fergusonii*, and other strains from the genera *Shigella*, *Citrobacter*, and *Salmonella*, are closely related (Fig. S1a,b in supporting information). The *E. fergusonii* strains used in the NCBI reference database have 99.73% sequence similarity with an *E. coli* strain. Furthermore, studies have also shown that there is a lack of monophyly between *Shigella* and *Escherichia coli* and among *Shigella* taxonomic groups^34,35^. This makes it clear why the average MinION read accuracy (89% in our study) would make it difficult to reliably distinguish related species, such as those within the *Enterobacteriaceae*, that share a high sequence similarity. Recently developed methods such as Metagenomic phylogenetic analysis (MetaPhlAn) for species-level profiling in large scale microbial community studies^36^ or Electronic probe Diagnostic for Nucleic acid Analysis (EDNA) to detect pathogens in metagenomics database^37^ might overcome these challenges and contribute in reliably detecting putative pathogens at species level. In addition, both of these techniques use species-specific markers, and can decrease the probability of both false positive and false negative profiling of microorganisms. When considering the implications of these findings for water quality analysis, Illumina sequencing with its shorter read length may result in false negative results, i.e. *E. coli* is present in the sample, but not detected at genus and species level. MinION sequencing with its longer read lengths, but lower accuracy, may result in underreported abundances (i.e. *E. coli*), and also false positives (i.e. *E. fergusonii* and other closely related species). Although both NGS methods for 16S rRNA amplicon sequencing, with their associated bioinformatics platforms, have short-comings when it comes to reliably establishing identities at species level, they can provide initial insight into the likely microbial community composition, which is not readily available from other analytical methods. While species level identities need to be interpreted with great caution, more reliable information is obtained at genus and family level, and can guide further investigations of microbial water quality. A combination of NGS for screening, and qPCR methods for validation, can thus help identify false negative or false positive results. When using total 16S rRNA, total coliforms, total *E. coli*, human *E. coli* and *Vibrio cholerae* marker gene primers to analyse the MOCK community, the results were consistent with the true composition of the MOCK community. For example, detection of *E. coli* by qPCR would help identify one of the *Enterobacteriaceae* species in the MOCK community, which Illumina NGS had classified only to family level. The absence of the cholera toxin excretion protein gene by qPCR would flag up a few MinION NGS reads mis-classified as *Vibrio cholerae* as potential false positives. For the water samples from the Kathmandu Valley, the Spearman rank correlations between various methods to target FIO were generally positive and significant in this study, in line with previous findings^38^. However, there were discrepancies between thermo-tolerant coliform bacteria by the plate count method, and those analysed with qPCR for several of the disinfected water samples. These discrepancies suggest that coliform bacteria were inactivated by the disinfection, but inactivated cells and/or their DNA were still present in the water. For example, DNA based methods such as qPCR and NGS (both Illumina and MinION) will not differentiate between viable and dead bacterial cells, or extracellular DNA, and therefore may have poor comparability with culture based techniques^18,31^, especially in disinfected waters. In such instances, the combined information of DNA based and culture based methods adds meaningful value to the water quality survey, because it suggests that some water samples were fit for consumption due to treatment (i.e. disinfection by the bottling company), but that the water sources were influenced by faecal contamination. Nucleic acid extraction methods incorporating PMA (propidium monoazide) can distinguish between active/live and dead bacteria, and may provide an avenue for the reliable detection of viable pathogens in future work^39^. Other minor discrepancies between various methods may also be due to method limitations (Fig. 6). The detection of bacteria from water with culture independent methods requires a sufficient amount of target DNA, and effective DNA extraction. However, rare species may not always be present in 100 mL of sample, and DNA extraction efficiency is not generally 100% and therefore can cause loss of target bacteria which are present in low amounts in the sampled water^19,40^. In addition, primers used for molecular methods can be too specific, and therefore some of the bacteria that can grow on the plate may still be missed with such methods. While a qPCR method can in theory amplify and detect a single target gene copy, samples with a low amount of target can show high variability and fall outside the linear range of the qPCR standard curve^41^. Despite of the several caveats discussed above, the methods used in this study are complementary, and if used in combination, they enable a fuller understanding the potential risks associated with different water sources than any method on its own (Fig. 6). For example, *Vibrio cholerae* MinION data from the Kathmandu Valley were well aligned with qPCR results targeting a marker gene for pathogenic *Vibrio cholerae* strains. These genes were generally detected only in the samples from tube wells (location 6, 7 and 8), which were also the only samples for which NGS with the MinION matched some reads to *Vibrio cholerae* at species level. The combined data gives a much higher level of confidence in the presence of potentially pathogenic *Vibrio cholerae* bacteria in these water samples, than NGS data on its own. The *Vibrio cholerae* hazard was not detected with Illumina NGS, and *Vibrio cholerae* MinION and *Vibrio cholerae* qPCR data were negatively correlated with the faecal *E. coli* plate count. Screening with MinION NGS can flag up such hazards and guide the choice of subsequent testing protocols, such as qPCR, to verify the hazard. In addition to the method development and cross-comparison we also wanted to demonstrate their applicability to comprehensive water quality monitoring in informal settlements. A discussion of chemical water quality parameters is provided as supporting information. WHO and Nepalese drinking water quality guidelines state that water intended for drinking or in the distribution system for drinking purposes should not contain faecal coliforms in a 100 mL sample^10,42^. The water from four locations in the Kathmandu Valley (one community tube well (location 8), spring water (location 9), stone spout (location 11) and one piped supply (location 12)), out of thirteen locations, was contaminated with culturable faecal coliforms, and therefore was not fit for consumption according to standard methods. The water from a spring (location 9) had the highest faecal *E. coli* concentration. These observations raise concerns, because springs are commonly used as a water source, also for the production of bottled or jar water and even tap water. Molecular methods also identified DNA from faecal bacteria in treated water samples (e.g., bacteria from coliform groups were detected in the water from locations 3, 12 and 13 when quantified with qPCR). 16S rRNA gene copy numbers were high in piped water from location 12, indicating a high amount of bacterial DNA in the tap water. Indicator gene copy numbers for the total coliforms were relatively higher as compared to other locations in the water sampled from location 9 (spring water), 10 (delivery truck) and 12 (piped water). Indicator genes for human *E. coli* pollution were only detected in water sampled from location 9 (spring water) and 12 (piped water). The molecular data raises strong concerns about the quality of spring water as a drinking water source, while total coliforms detected by plate count in one sample from the piped supply (location 12, also positive for faecal coliforms by plate count), and delivery truck water (location 10) raises strong concerns about the effectiveness of water treatment, as previously reported by other studies^2,6,8^. Clearly, water treatment needs to be more robust, if water sources such as springs have very poor microbial quality. The MinION NGS and qPCR *Vibrio cholerae* data also raises serious concerns about the safety of water from shallow tube wells (location 6, 7 and 8). Knowledge of common waterborne disease-causing agents prevailing in the sampling region (Table S11 in supporting information) can provide additional context for the interpretation of microbial water quality data. None of the MinION NGS reads from locations 2, 3 and 4 matched pathogens known to cause disease in the Kathmandu Valley, while in all the other locations, at least one such pathogen was present in the water samples according to MinION NGS results. In the water sample from location 6 (tube well), putative pathogens like *Campylobacter, Clostridium, Salmonella, Shigella* and *Vibrio cholera*e were detected, while *Clostridium botulinum, Escherichia coli, Salmonella enterica* and *Shigella* were detected in the piped water sample from location 12. A significant number of reads were matched by FASTQ 16S workflow of ONT at species level to bacterial agents mentioned in the approved list of biological agents by Health and Safety Executive (HSE), UK^43^. While the majority of OTUs in Illumina data were not resolved at species level, the pattern for the relative abundance of *Pseudomonas* and *Erysipelothrix* across sampling locations was consistent with the MinION NGS data for putative pathogen species within these genera, showing an unusually high number of MinION reads matched to *Pseudomonas aeruginosa* in location 4, and *Erysipelothrix rhusiopathiae* in location 6. The relative abundance of MinION reads matched to putative pathogens on the HSE list was on average higher in water from tube wells compared to other water types. As mentioned earlier, due to the risk of false positive identifications at species level with the MinION NGS method, additional tests using qPCR and culturing techniques (to check for viability) would be required to confirm the presence of these putative pathogens in the water samples. MinION NGS data is helpful in providing direction for such future work. With careful interpretation and cross-validation of results, NGS data adds significant value to water quality assessments, as demonstrated in this survey. As a low-cost, portable NGS tool generating long sequencing reads, the MinION of ONT is especially promising. The significant rank correlations between the relative abundances of *Bacteroides, Prevotella*, *Enterobacteriaceae* and all other putative pathogenic genera determined by MinION and Illumina NGS suggest that the portable, memory-stick sized MinION provides a valid alternative to the Illumina platform, which is that currently most widely used for NGS community-based microbial water quality monitoring and environmental surveillance^22,25^. However, further improvements of protocols, nanopore technology, and bioinformatics workflows are needed to improve read accuracy and avoid false assignment of reads to species. For instance, *Calus et al*. have recently developed a workflow for MinION sequencing to estimate the diversity of MOCK communities with average sequence accuracy of 99.5%. While their library preparation protocol using rolling circle amplification and enhanced bioinformatics dramatically improved the accuracy of MinION NGS, the total number of accepted reads generated per sequencing run was reduced accordingly^44^. The MinION NGS has already been applied in the field of public and animal health, and water security to identify specific pathogenic strains^24,45–48^. *Theuns et al*. successfully used MinION NGS as a diagnostic tool for porcine viral enteric disease and revealed porcine kobuvirus as the main enteric disease causing virus in swine^45^. *Rames and Macdonald* were able to detect Enteroviruses (EV) in wastewater (WW) samples with MinION sequencing, after spiking EV into the WW prior to sequencing^46^.

## Methods

### Water sampling locations

Water samples in this study were collected from the most common water sources used by residents in the Kathmandu Valley. These comprised shallow tube wells, deep boreholes, stone spouts (dhunge dhara, a traditional ornamental spring), piped water, water supplied by delivery truck, bottled drinking water, and jar water (i.e. commercially available large containers of water used for drinking and cooking). Water samples were collected from 13 sites across the Kathmandu Valley. The locations of these water sources are presented in Fig. 3 and described in Table 1.

### Collection of water samples for analysis

From each of the sources, 3 litres of water were collected in sterile 1 litre bottles. During sampling, the lid of the sterile bottle was opened and closed aseptically, and bottles were rinsed thoroughly with water from the same source before sample collection. From the piped water supply and stone spout, water was allowed to flow directly into the sterile bottles. For tube wells and boreholes, the water was pumped using a vacuum pump and polyethylene pipe. Sufficient purging was performed to prevent the collection of standing water already present in the pipe. Prior to collecting the water, the outlet of the tube was sterilized using 70% ethanol and flamed with a burning cotton swab, and water was then allowed to flow directly into the sterile bottle. The bottled drinking water and jar water were bought from local vendors. The sample bottles were stored in an insulated cold-box with ice packs inside and transported to the laboratory to be processed within two hours.

### Microbial water quality analysis

Total coliform and faecal E. coli bacteria were determined by membrane filtration in duplicate at Nobel College (NC), Kathmandu, Nepal, following Standard Methods for the Examination of Water and Wastewater^49^. Different volumes of water (250 mL up to 2 L) were filtered through 0.22 µm membranes (Sartorius UK Limited, Surrey, UK) depending on the turbidity of the water from each sampling site and were immediately stored at −20 °C to preserve DNA for subsequent molecular microbiology. The molecular work was then conducted at Newcastle University (NU), Newcastle upon Tyne, UK. The total DNA from prokaryotic biomass retained by the membrane was extracted using a PowerWater® DNA Isolation Kit as per the manufacturer’s instructions (QIAGEN, Crawley, UK). DNA purity and concentration were determined using a DS-11 FX + Spectrophotometer/Fluorometer (DeNovix, Delaware, USA). 30 ng of DNA was used to build a 16S rRNA prokaryote gene sequencing library for nanopore sequencing using a 16S Barcoding kit (SQK-RAB204 from Oxford Nanopore Technologies (ONT), Oxford, UK) as per the manufacturer’s instructions and loaded onto a MinION sequencing apparatus flow cell (R9.4.1, FLO-MIN106). Table S12 in supporting information lists the primers used for 16S rRNA amplicon sequencing with the MinION. The flow cell was placed into the MinION sequencing device and controlled using ONT’s MinKNOW software. The sequencing run was performed for 48 hrs. The raw reads (i.e. HDF5 raw signals) were base-called (i.e. converting the electrical signals generated by a DNA or RNA strand passing through the nanopore into the corresponding base sequence of the strand) with Albacore (Version; v2.3.3) software (ONT, Oxford, UK) producing.fastq files. Base-called data were uploaded to the EPI2ME interface, a platform for cloud based analysis of MinION data. Data interpretation was performed with the FASTQ 16S workflow (for quality filtering, a quality score ≥7 was used). The FASTQ 16S workflow [rev.2.1.1] analysis revealed the taxonomic classification of base-called reads along with their frequency, which ultimately was used to estimate the relative abundance of the putative human pathogens mentioned in the review by the UK Health and Safety Executive (HSE 2013).

Further, the extracted DNA were also sequenced (paired end sequencing; 2 × 250 bp) in duplicate with an Illumina Miseq platform (NU-OMICS, Northumbria University, UK) using the primer set targeting the V4 region of the bacterial 16S rRNA (Table S12) as described elsewhere^50^. The amplicon data from Illumina were processed using an open source software package: Quantitative Insights Into Microbial Ecology, QIIME 2 (https://qiime2.org/). Denoising and dereplication of pair end sequencing, including chimera removal and trimming of reads based on positional quality scores, were performed using the Divisive Amplicon Denoising Algorithm 2 (DADA2)^51^. The VSEARCH clustering method was used to cluster the quality-filtered sequences into ASVs (amplicon sequencing variants) that were converted into OTUs (operation taxonomic units), with a threshold of 97% identity^52^. Finally, taxonomy of each OTU was assigned by matching to the GreenGenes database (v13_8), based on a naïve Bayesian classifier with default parameters.

In order to assess bias and errors in both NGS methods, a MOCK community (i.e. DNA mixture of known bacterial species in fixed proportion, see Table S1) provided by Zymo Research (Catalogue number: D6306), Freiburg, Germany, was also included in the sequencing runs in triplicate, and processed and analysed in the same way as the samples. To assist interpretation of NGS results for the MOCK community, we constructed a phylogenetic tree and identity matrix using the 16S rRNA gene sequences for different strains of *E. coli, E. fergusonii*, closely related other strains from the genera *Shigella, Citrobacter*, and *Salmonella*, and a more distant strain (*Enterococcus faecalis*) recorded in the NCBI reference database (Fig. S1a,b). It should be noted that the FASTQ 16S workflow in EPI2ME uses the NCBI 16S rRNA database as a reference database for taxonomic classification.

Real time PCR assays (qPCR) were performed to quantify the number of target genes on a BioRad CFX C1000 system (BioRad, Hercules, CA USA) using the primers shown in Table S12. For quantification of the target genes, 2 μl template DNA was used in a reaction mixture containing 5 μL 2 × SsoAdvanced Universal SYBR Green Supermix (Bio-Rad), 500 nmol L^−1^ of each forward and reverse primer, and molecular grade H_2_O (Invitrogen, Life Technologies, Paisley, UK) to a final volume of 10 μL. Reaction conditions for quantification of each target gene were 98 °C for 3 min (1x), then 98 °C for 15 s, and the Primer Annealing Temperature (Ta) for 60 s (Table S12) (40 cycles). Standard curves were constructed using the synthesized nucleotide sequence of the target gene (Invitrogen, Life Technologies, Paisley, UK), and generated every time a qPCR analysis was performed, in parallel with the amplification of test samples. Serial dilution (10-fold) of the standards was performed to obtain standard solutions in the range of 10^8^–10^1^ target gene copies/μL. All samples were run in triplicate and molecular grade H_2_O replaced template in control reactions. To avoid inhibitor effects, DNA samples were diluted to a working solution of 5 ng/uL.

### Chemical water quality analysis

Chemical water quality analysis methods are described in supporting information.

### Statistical analysis

A Spearman rank correlation analysis between different microbial water quality indicators determined by standard plate counting method, qPCR and next generation sequencing (NGS) approaches was performed in R-studio.

## Supplementary information

Supplementary information

## Data Availability

Data created during this research are openly available at DOI 10.25405/data.ncl.9693533. Please contact Newcastle Research Data Service at rdm@ncl.ac.uk for access instructions.
