# Supplementary material for: A comparative assessment of conventional and molecular methods, including MinION nanopore sequencing, for surveying water quality
Source: Sci Rep. 2019 Oct 31;9:15726. doi: 10.1038/s41598-019-51997-x (PMC6823499; doi:10.1038/s41598-019-51997-x)
Supplement: Supplementary file 1 — Supplementary information [file 41598_2019_51997_MOESM1_ESM.pdf]

Supporting Information

A comparative assessment of conventional and molecular methods, including MinION nanopore sequencing, for surveying water quality

Kishor Acharya[†], *Santosh Khanal*[‡], *Kalyan Pantha*[# ǂ], *Niroj Amatya*[¥ ǂ], *Russell J. Davenport*[†], *David Werner*[†*]

[†] School of Engineering, Newcastle University, Newcastle upon Tyne, NE1 7RU, United Kingdom

[‡] Department of Pharmacology, School of Medicine, University of Colorado, Aurora, Colorado 80045, USA

[#] Group for Rural Infrastructure Development, Wise use House, Jwagal, Lalitpur, Nepal

[ǂ] Faculty of Chemistry, University Duisburg-Essen, Universitätsstr. 5, D-45141 Essen, Germany

[¥]Department of Medical Microbiology, Nobel College, Pokhara University, Kathmandu, Nepal

[*]Corresponding author: david.werner@ncl.ac.uk

Chemical water quality assessment for different water sources in the Kathmandu Valley

Methods

Grab water samples were analysed for pH, dissolved oxygen, electrical conductivity, and temperature on-site using a portable probe from EXTECH INSTRUMENTS (Boston, USA). For chemical analysis, water samples were filtered on-site through 0.22 µm PVDF syringe filters (Gilson Scientific Ltd, Dunstable UK), collected in two 50 mL sterile polyethylene vials and then the vials were frozen for analysis at NU. HACH cuvette test kits were used to measure nitrite (NO_2^--N), nitrate (NO_3^--N), ammonia (NH_3-N), and fluoride (F^-) in the filtered samples, following the manufacturer's instructions (HACH LANGE LTD, Manchester, UK). Similarly, alkalinity was measured using a HACH digital titrator, following the manufacturer's instructions (HACH LANGE LTD, Manchester, UK). Analysis of filtered metals was undertaken using a Varian Vista-MPX ICP-OES or Agilent 770 Series ICP-MS, as appropriate for the metal concentration. Anion analysis was conducted using a Dionex DX320 ion chromatograph. Blanks and standards were used throughout and duplicate samples were analysed for HACH cuvette test. For quality assurance, charge balances were calculated to check results for electric neutrality considering Na^+, K^+, Mg^{2+}, Ca^{2+}, HCO_3^-, Cl^-, NO_3^- and SO_4^{2-} charges [1].

Results

In addition to microbial water quality it is important to not overlook chemical water quality issues. **Table S8** compares concentrations of different relevant ions in the water from the 13 different sources. The Nepali national drinking-water quality standard and the WHO guideline for nitrate concentration in drinking water is 50 mg/L, [2,3]; water samples from two sources, the stone spout (location 11) and a tube well (location 8), contained concentrations at or slightly above this guideline value. Chloride, nitrite and fluoride concentrations were

below WHO guidelines of 250 mg/L, 3 mg/L and 1.5 mg/L, respectively [2], in all the water samples (Nepali standard for chloride, fluoride and nitrite are not available), while sulphate concentrations were below the Nepali standard of 250 mg/L in all the waters. The Nepali standard for ammonia of 1.5 mg/L was violated in one water sample (location 7; tube well) with a concentration of 1.779 mg/L. The pH of water samples ranged from 6.2 to 8.16 (**Table S9**). The Nepali national water quality guideline requires pH to be between 6.5 and 8.5 [3]. The pH was < 6.5 in three of the sources (location 7, 8 and 13). None of the water sources violated the Nepali standard of 1500 uS/cm specific conductance (**Table S9**).

Table S10 shows concentrations of different metals analysed in the water from the 13 sources. A study by *Shrestha et. al.* reported heavy metals contamination in deep groundwater sources in the Kathmandu Valley, especially arsenic, zinc, lead, iron and manganese [4]. However, none of the filtered samples collected in this study exceeded the Nepali water-quality standard for arsenic, zinc and lead of 50 ug/L, 3 mg/L and 0.01 mg/L, respectively. Exceedance of the Nepali standard for iron of 0.3 mg/L (WHO guideline for iron not available) was observed in the water from two sources; location 5 (deep borehole) and location 7 (tube well). Likewise, samples from three sources; location 5 (deep borehole), location 6 (tube well) and location 7 (tube well), violated the WHO (0.1 mg/L) drinking water quality guideline for manganese. Locations 5 and 7 violated the Nepali manganese standard (0.2 mg/L). The WHO and Nepali standard for aluminium of 0.2 mg/L was violated in one water sample (location 4; tube well) with a concentration of 0.214 mg/L. Concentrations of all other metals, including Ca, Mg, Na, K, Ba, Cu, Cd, Sb, Si, Ni, Cr and Sr were well below the Nepali and WHO guidelines.

(a)

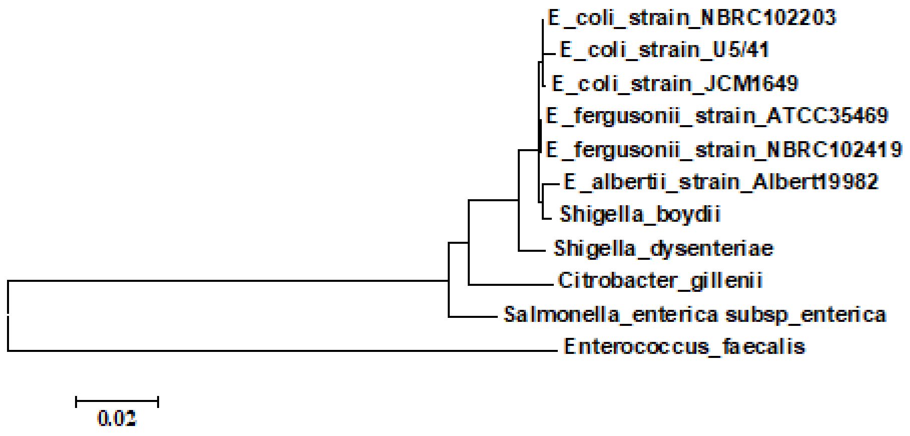

(b)

Percent Identity Matrix - created by Clustal2.1

1: Enterococcus_faecalis	100.00	75.28	76.72	76.38	75.50	75.82	76.03	75.58	76.14	75.72	76.87
2: Citrobacter_gillenii	75.28	100.00	96.07	96.23	95.31	95.72	95.35	96.18	95.61	96.25	96.40
3: Salmonella_enterica	76.72	96.07	100.00	97.11	96.28	96.92	97.16	96.86	97.33	96.93	96.40
4: Shigella_dysenteriae	76.38	96.23	97.11	100.00	98.05	98.31	98.65	98.77	98.99	98.84	98.92
5: E_coli_strain_U5/41	75.50	95.31	96.28	98.05	100.00	98.18	98.39	98.53	98.83	98.81	99.37
6: E_albertii_strain_Albert19982	75.82	95.72	96.92	98.31	98.18	100.00	99.20	98.98	99.13	99.05	99.28
7: Shigella_boydii	76.03	95.35	97.16	98.65	98.39	99.20	100.00	99.32	99.60	99.18	99.37
8: E_fergusonii_strain_NBRC102419	75.58	96.18	96.86	98.77	98.53	98.98	99.32	100.00	99.59	99.32	99.73
9: E_fergusonii_strain_ATCC35469	76.14	95.61	97.33	98.99	98.83	99.13	99.60	99.59	100.00	99.59	99.73
10: E_coli_strain_NBRC102203	75.72	96.25	96.93	98.84	98.81	99.05	99.18	99.32	99.59	100.00	99.91
11: E_coli_strain_JCM1649	76.87	96.40	96.40	98.92	99.37	99.28	99.37	99.73	99.73	99.91	100.00

Figure S1 (a) Phylogenetic distance tree (Neighbour-Joining) generated with 16S rRNA sequences of *E coli*, *E fergusonii*, closely related other strains and a distant strain. The 16S rRNA sequences were obtained from the NCBI 16S rRNA database. (b) The percent identity matrix of the 16S rRNA sequences used for creating the phylogenetic distance tree.

Table S1 Actual composition of different bacterial species present in the Mock Community based on genomic DNA[5].

**Microorganisms**	**Percentage DNA composition (%)**	**16S rRNA gene composition (%)**
Listeria monocytogenes	12	14.1
Pseudomonas aeruginosa	12	4.2
Bacillus subtilis	12	17.4
Escherichia coli	12	10.1
Salmonella enterica	12	10.4
Lactobacillus fermentum	12	18.4
Enterococcus faecalis	12	9.9
Staphylococcus aureus	12	15.5
Saccharomyces cerevisiae	2	na
Cryptococcus neoformans	2	na

Table S2 MinION 16S rRNA sequencing result for the MOCK Community at species level. The 16S rRNA amplicon sequencing was performed in triplicate. The species highlighted in blue are the actual bacteria who's DNA are present in the MOCK community.

**Species**	**Average number of reads**	**Standard deviation**	**16S rRNA gene percentage (%)**	**Standard deviation**
Lactobacillus fermentum	13446.33	3666.82	9.25	0.13
Staphylococcus aureus	12443.67	4241.45	8.47	0.54
Enterococcus faecalis	7831.33	2077.18	5.39	0.10
Salmonella enterica	7177.33	2420.26	4.89	0.28
Bacillus mojavensis	5848.00	1884.89	3.99	0.18
Pseudomonas aeruginosa	5373.33	1811.16	3.66	0.32
Bacillus halotolerans	4772.33	1403.69	3.27	0.06
Bacillus subtilis	4509.00	1242.45	3.10	0.05
Listeria innocua	3211.00	1002.29	2.19	0.07
Bacillus vallismortis	1675.67	305.03	1.17	0.12
Staphylococcus simiae	1669.00	465.84	1.15	0.06
Listeria seeligeri	1412.00	370.07	0.97	0.04
Listeria monocytogenes	1096.33	68.09	0.78	0.15
Escherichia fergusonii	940.00	198.87	0.65	0.06
Bacillus atrophaeus	858.67	251.42	0.59	0.03
Listeria ivanovii	775.00	288.97	0.52	0.05
Lactobacillus gastricus	746.33	246.02	0.51	0.04
Bacillus amyloliquefaciens	584.00	137.07	0.40	0.02
Staphylococcus capitis	602.67	192.27	0.41	0.02
Shigella sonnei	449.67	93.35	0.31	0.06
Staphylococcus haemolyticus	452.67	117.50	0.31	0.02
Bacillus altitudinis	456.00	89.54	0.32	0.04
Shigella flexneri	421.33	143.54	0.29	0.02
Staphylococcus hominis	339.33	88.93	0.23	0.00
Staphylococcus auricularis	211.00	24.52	0.15	0.04

Table S2 Continued

Citrobacter koseri	250.67	34.30	0.18	0.02
Escherichia coli	247.33	31.79	0.17	0.02
Bacillus nematocida	257.00	45.90	0.18	0.02
Bacillus velezensis	238.00	55.07	0.16	0.01
Staphylococcus caprae	251.33	46.06	0.18	0.02
Bacillus nakamurai	213.33	13.43	0.15	0.03
Lactobacillus equigenerosi	221.67	39.50	0.16	0.02
Staphylococcus saccharolyticus	185.33	57.83	0.13	0.01
Listeria marthii	190.33	33.50	0.13	0.02
Pseudomonas otitidis	182.67	73.16	0.12	0.02
Bacillus licheniformis	177.33	44.74	0.12	0.01
Staphylococcus petrasii	148.00	18.33	0.10	0.01
Listeria grayi	147.33	41.79	0.10	0.01
Enterococcus wangshanyuanii	132.00	14.93	0.09	0.01
Bacillus cereus	143.00	28.62	0.10	0.01
Bacillus shackletonii	153.00	24.25	0.11	0.02
Enterococcus rivorum	163.33	42.02	0.11	0.02
Pseudomonas resinovorans	144.67	48.09	0.10	0.01
Staphylococcus sciuri	123.67	9.07	0.09	0.02
Enterococcus faecium	147.67	30.57	0.10	0.02
Staphylococcus delphini	121.33	21.39	0.08	0.01
Lactobacillus gorillae	137.33	35.92	0.09	0.01
Bacillus stratosphericus	95.33	8.02	0.07	0.02
Lactobacillus reuteri	124.67	59.00	0.08	0.02
Bacillus siamensis	109.00	28.93	0.08	0.00
Bacillus zhangzhouensis	99.33	6.81	0.07	0.01
Enterococcus hirae	106.33	27.65	0.07	0.00
Bacillus firmus	111.00	27.87	0.08	0.00
Staphylococcus piscifermentans	95.67	13.87	0.07	0.01
Macrococcus caseolyticus	99.67	15.89	0.07	0.01
Listeria aquatica	79.67	9.02	0.06	0.02
Staphylococcus epidermidis	115.33	42.44	0.08	0.01
Bacillus pumilus	106.33	24.58	0.07	0.01
Bacillus safensis	109.67	27.50	0.08	0.01
Pseudomonas alcaligenes	82.67	18.77	0.06	0.01
Lactobacillus mucosae	112.00	42.53	0.08	0.01
Bacillus swezeyi	107.67	31.88	0.07	0.01
Bacillus haynesii	72.33	2.08	0.05	0.01
Klebsiella pneumoniae	96.67	25.54	0.07	0.01
Staphylococcus devriesei	89.33	18.61	0.06	0.01
Bacillus acidicola	81.67	10.50	0.06	0.01
Pseudomonas stutzeri	85.33	19.86	0.06	0.00

Table S2 Continued

Staphylococcus massiliensis	80.67	11.93	0.06	0.02
Enterococcus sulfureus	94.33	26.41	0.06	0.01
Escherichia marmotae	86.33	20.98	0.06	0.00
Lactobacillus ingluviei	88.67	31.56	0.06	0.00
Enterobacter cloacae	69.00	5.29	0.05	0.01
Enterococcus saccharolyticus	93.67	35.53	0.06	0.01
Bacillus pseudofirmus	84.00	32.05	0.06	0.01
Staphylococcus kloosii	75.33	24.21	0.05	0.00
Bacillus ginsengihumi	66.67	5.69	0.05	0.01
Listeria floridensis	79.67	22.50	0.05	0.00
Pseudomonas pseudoalcaligenes	60.33	13.50	0.04	0.01
Bacillus sporothermodurans	65.00	12.17	0.05	0.00
Staphylococcus carnosus	65.33	7.09	0.05	0.01
Staphylococcus felis	62.33	4.73	0.04	0.01
Shigella dysenteriae	65.67	17.67	0.05	0.00
Bacillus cohnii	64.00	11.00	0.04	0.01
Pseudomonas argentinensis	48.33	3.51	0.04	0.01
Enterococcus termitis	56.67	5.77	0.04	0.01
Citrobacter farmeri	71.33	32.01	0.05	0.01
Bacillus songklensis	67.67	20.01	0.05	0.01
Bacillus paralicheniformis	48.00	10.00	0.04	0.01
Staphylococcus simulans	53.00	11.27	0.04	0.00
Escherichia albertii	67.33	19.14	0.05	0.01
Bacillus herbersteinensis	39.00	6.24	0.03	0.01
Shigella boydii	50.67	10.79	0.04	0.00
Bacillus carboniphilus	49.67	3.51	0.04	0.01
Bacillus halmapalus	52.67	10.26	0.04	0.00
Bacillus luciferensis	38.33	5.51	0.03	0.01
Lactobacillus siliginis	42.33	3.51	0.03	0.01
Bacillus mycoides	52.33	17.21	0.04	0.00
Bacillus galliciensis	49.67	15.95	0.03	0.00
Pseudomonas jinjuensis	41.33	2.08	0.03	0.01
Bacillus thuringiensis	35.00	3.46	0.02	0.01
Bacillus aquimaris	43.00	8.89	0.03	0.00
Listeria weihenstephanensis	35.33	11.02	0.02	0.01
Staphylococcus chromogenes	50.33	14.01	0.03	0.01
Bacillus flexus	43.00	10.58	0.03	0.00
Enterococcus pseudoavium	43.00	7.55	0.03	0.00
Shimwellia blattae	42.33	10.21	0.03	0.00
Bacillus cytotoxicus	36.00	11.14	0.02	0.00
Bacillus isabeliae	34.33	4.51	0.03	0.01
Kosakonia sacchari	32.33	10.60	0.02	0.01
Enterococcus avium	37.33	9.29	0.03	0.00

Table S2 Continued

Bacillus gottheilii	36.33	5.86	0.03	0.00
Cronobacter sakazakii	25.33	5.86	0.02	0.01
Pseudomonas guguanensis	37.67	5.77	0.03	0.01
Bacillus aerius	49.33	16.26	0.03	0.01
Staphylococcus gallinarum	39.00	8.54	0.03	0.00
Bacillus xiamenensis	39.00	8.19	0.03	0.01
Bacillus zhanjiangensis	31.33	2.31	0.02	0.00
Pseudomonas mendocina	32.00	2.00	0.02	0.01
Bacillus deserti	29.67	2.52	0.02	0.00
Enterococcus moraviensis	41.67	17.47	0.03	0.01
Staphylococcus lugdunensis	31.33	4.51	0.02	0.01
Enterococcus dispar	32.00	6.24	0.02	0.00
Bacillus mediterraneensis	26.67	3.06	0.02	0.00
Bacillus timonensis	29.67	3.51	0.02	0.01
Enterococcus haemoperoxidus	32.00	6.00	0.02	0.00
Enterococcus canis	41.67	16.04	0.03	0.01
Staphylococcus xylosus	26.33	3.21	0.02	0.00
Staphylococcus stepanovicii	30.67	6.11	0.02	0.00
Staphylococcus cohnii	27.33	3.51	0.02	0.00
Bacillus fastidiosus	23.67	7.02	0.02	0.00
Enterococcus asini	25.67	6.43	0.02	0.01
Lactobacillus vaccinostercus	33.00	14.00	0.02	0.00
Listeria rocourtiae	28.00	4.58	0.02	0.00
Others	58901.33	15512.66	40.59	0.42
**Total number of reads**	**145392**	**39928.05**		

Table S3 Plate count results for Fecal *E.coli* and Total coliform in the water samples from 13 different sites. Data points are an average of duplicate biological samples and the number in parentheses indicates the standard deviation. Where required, the dilution was performed with normal saline solution (NS). Two blanks; membrane filter (MF) and NS, were also tested for Fecal *E.coli* and Total coliform in duplicate.

**Sample Site ID**	**1**	**2**	**3**	**4**	**5**	**6**	**7**	**8**	**9**	**10**	**11**	**12**	**13**	**Blank (MF)**	**Blank (NS)**
**Faecal *E coli* (CFU/100 mL) WHO and Nepalese guideline value: 0 CFU/100 mL**	0	0	0	0	0	0	0	0.5 (0.7)	215 (134.4)	0	1.5 (0.7)	3 (1.4)	0	0	0
**Total Coliform (CFU/100 mL) WHO and Nepalese guideline value: 0 CFU/100 mL**	0	0	0	0	0	330 (14.14)	>20000	>200	>2000	>2000	88.5 (2.12)	39.5 (14.85)	0	0	0

Table S4 Relative abundances of different putative genera estimated from 16S rRNA reads sequenced with the Illumina NGS platform. The numbers are an average of duplicate samples and the numbers in parenthesis indicate the standard deviation. n.d indicated not detected.

**Location**	***Clostridium***	***Vibrio***	***Legionella***	***Streptococcus***	***Prevotella***	***Bacteroides***	***Pseudomonas***	**Total Reads**
**Location 2**	1.70E-02 (1.20E-02)	2.28E-02 (4.62E-03)	n.d	6.98E-04 (2.32E-04)	7.59E-04 (7.61E-04)	n.d	2.14E-03 (3.25E-04)	42258 (4162)
**Location 3**	n.d	8.38E-04 (8.32E-05)	2.96E-05 (4.19E-05)	4.50E-04 (8.61E-05)	3.24E-04 (8.11E-05)	n.d	2.22E-04 (3.24E-05)	130401 (63480)
**Location 4**	1.08E-02 (8.03E-04)	9.48E-05 (2.79E-07)	n.d	6.49E-04 (8.61E-06)	4.60E-04 (8.85E-05)	1.04E-04 (1.47E-04)	1.68E-02 (3.52E-03)	79055 (6469)
**Location 5**	3.69E-03 (2.01E-03)	2.18E-04 (1.36E-04)	6.17E-05 (8.72E-05)	n.d	7.58E-05 (4.38E-05)	n.d	n.d	77394 (51602)
**Location 6**	1.82E-02 (1.61E-03)	1.90E-04 (3.86E-05)	4.75E-04 (3.02E-05)	n.d	n.d	1.60E-05 (2.26E-05)	1.95E-04 (5.85E-05)	139606 (37060)
**Location 7**	1.42E-02 (7.22E-03)	1.60E-05 (2.26E-05)	n.d	n.d	n.d	3.16E-04 (4.12E-05)	3.76E-05 (5.31E-05)	144883 (11351)
**Location 8**	2.10E-02 (1.04E-02)	5.69E-04 (1.75E-05)	6.04E-04 (1.12E-04)	n.d	n.d	6.39E-05 (5.46E-05)	4.85E-04 (1.04E-04)	122936 (16706)
**Location 9**	9.39E-02 (1.40E-02)	n.d	3.31E-04 (2.21E-05)	n.d	2.09E-03 (4.43E-04)	1.38E-03 (1.52E-04)	1.34E-02 (4.83E-05)	115934 (64222)
**Location 10**	2.25E-01 (4.66E-02)	8.38E-05 (1.27E-05)	4.46E-04 (4.88E-05)	4.22E-05 (5.96E-05)	4.53E-03 (1.29E-03)	3.51E-03 (5.70E-04)	2.99E-02 (7.50E-04)	112765 (23757)
**Location 11**	1.59E-02 (3.21E-03)	1.57E-03 (1.79E-04)	2.67E-04 (3.78E-04)	5.55E-05 (7.85E-05)	1.59E-03 (5.97E-04)	4.17E-04 (2.35E-04)	8.15E-04 (5.75E-04)	33930 (7285)
**Location 12**	2.33E-01 (3.29E-01)	2.92E-04 (2.07E-04)	1.32E-04 (1.86E-04)	2.83E-03 (9.55E-04)	2.75E-02 (6.75E-03)	5.22E-03 (4.29E-05	3.63E-03 (2.03E-03)	156004 (48730)
**Location 13**	n.d	3.96E-04 (1.73E-04)	3.68E-03 (2.65E-04)	n.d	2.76E-04 (2.38E-06)	6.80E-05 (2.63E-05)	4.21E-04 (3.72E-05)	115666 (49772)

Table S5 Relative abundances of different putative genera estimated from 16S rRNA reads sequenced with the MinION NGS platform. n.d indicated not detected.

**Location**	***Clostridium***	***Vibrio***	***Legionella***	***Streptococcus***	***Prevotella***	***Bacteroides***	***Pseudomonas***
**Location 2**	6.61E-04	3.21E-03	n.d	5.19E-04	n.d	n.d	1.39E-03
**Location 3**	2.64E-05	2.64E-05	4.40E-05	2.20E-04	1.76E-05	n.d	5.02E-04
**Location 4**	1.44E-04	1.29E-05	2.14E-05	4.40E-04	3.22E-05	6.43E-06	8.33E-02
**Location 5**	4.08E-05	3.50E-05	1.28E-04	4.08E-05	3.50E-05	1.17E-05	6.94E-04
**Location 6**	6.42E-04	6.52E-05	1.13E-03	1.36E-04	2.77E-05	6.91E-05	7.29E-04
**Location 7**	2.21E-04	9.75E-05	1.28E-04	3.75E-05	1.50E-05	3.38E-05	3.51E-03
**Location 8**	7.95E-04	1.32E-04	1.84E-03	1.54E-04	1.72E-05	2.29E-05	1.60E-03
**Location 9**	1.47E-03	6.51E-05	1.38E-03	1.86E-04	2.86E-04	2.17E-04	1.74E-02
**Location 11**	4.49E-03	1.88E-04	8.26E-03	2.37E-04	6.43E-04	3.16E-04	1.98E-02
**Location 12**	5.30E-05	1.44E-04	1.98E-03	4.00E-03	9.44E-03	3.91E-03	5.23E-04
**Location 13**	n.d	4.55E-05	3.52E-03	2.27E-05	3.03E-05	3.79E-05	3.93E-02

Table S6 Results of the Spearman rank correlation analysis (n = 12) between faecal indicator bacterial families or genera across all the water samples analysed with Illumina (Table S4) and MinION (Table S5).

**Putative pathogenic genera or family**	**Spearman rank Correlation coefficient**	**p-value**
Clostridium_MinION vs Clostridium_Illumina	0.96	< 0.05
Legionella_MinION vs Legionella_Illumina	0.82	< 0.05
Pseudomonas_MinION vs Pseudomonas_Illumina	0.51	< 0.05
Streptococcus_MinION vs Streptococcus_Illumina	0.89	< 0.05
Vibrio_MinION vs Vibrio_Illumina	0.52	< 0.05
Bacteroides_MinION vs Bacteroides_Illumina	0.84	< 0.05
Prevotella_MinION vs Prevotella_Illumina	0.66	< 0.05
Enterobacteriaceae_MinION vs Enterobacteriaceae_Illumina	0.81	< 0.05

Table S7 16S rRNA gene sequencing reads from MinION NGS platform matching the putative pathogens listed by HSE [6], and total number of reads per samples are reported in last row.

**Potential Pathogens[6]**	**Sampling Site ID**										
	2	3	4	5	6	7	8	9	11	12	13
Actinobacillus actinomycetemcomitans	0	0	0	0	0	0	0	0	0	0	0
Actinomadura madurae	0	0	0	0	1	0	0	0	0	1	0
Actinomadura pelletieri	0	0	0	0	0	0	0	0	0	2	0
Actinomyces gerencseriae	0	0	0	0	0	0	0	0	0	0	0
Actinomyces israelii	0	0	0	0	0	0	0	0	0	1	0
Actinomyces pyogenes	0	0	0	0	0	0	0	0	0	0	0
Arcanobacterium haemolyticum	0	0	0	0	1	0	0	1	0	2	0
Arcanobacterium pyogenes	0	0	0	0	0	0	0	0	0	0	0
Arcobacter butzleri	0	0	0	0	0	0	0	0	0	0	0
Bacillus anthracis	0	0	0	0	0	0	0	0	0	0	0
Bacillus cereus	0	0	0	0	0	0	2	0	4	0	3
Bacteroides fragilis	0	0	0	0	0	1	0	0	1	10	0
Bartonella bacilliformis	1	0	4	0	2	0	2	4	0	0	0
Bartonella quintana	0	0	0	0	0	0	0	0	0	0	0
Bordetella bronchiseptica	0	0	0	2	23	2	0	0	0	0	0
Bordetella parapertussis	0	2	0	1	40	6	0	0	2	0	5
Bordetella pertussis	0	0	0	0	1	0	0	0	0	0	1
Borrelia burgdorferi	0	0	0	0	0	0	0	0	0	0	0
Borrelia duttonii	0	0	0	0	0	0	0	0	0	0	0
Borrelia recurrentis	0	0	0	0	0	0	0	0	0	0	0
Brucella abortus	0	0	309	1	0	0	0	0	0	0	0
Brucella canis	0	0	185	0	1	0	0	1	0	0	2
Brucella melitensis	0	1	10	0	0	0	0	0	0	0	0
Brucella suis	0	0	17	0	0	0	0	0	0	0	0
Burkholderia cepacia	0	0	0	2	1	1	1	2	0	0	0

Table S7 Continued

Burkholderia mallei	0	0	0	0	0	0	0	0	0	0	0
Burkholderia pseudomallei	0	0	0	0	0	0	0	0	0	0	0
Campylobacter fetus	0	0	0	0	2	0	0	0	0	0	0
Campylobacter jejuni	0	0	0	0	3	0	0	0	1	0	0
Cardiobacterium hominis	0	0	0	0	0	0	0	0	0	0	0
Chlamydophila pneumoniae	0	0	0	0	0	0	0	0	0	0	0
Chlamydophila psittaci	0	0	0	0	0	0	0	0	0	0	0
Chlamydophila trachomatis	0	0	0	0	0	0	0	0	0	0	0
Clostridium botulinum	0	0	0	0	3	0	0	0	1	1	0
Clostridium perfringens	0	0	1	0	1	0	1	4	0	3	0
Clostridium tetani	0	0	0	0	0	0	0	0	0	0	0
Corynebacterium diphtheriae	0	7	0	0	1	0	0	0	0	0	0
Corynebacterium haemolyticum	0	0	0	0	0	0	0	0	0	0	0
Corynebacterium minutissimum	0	0	1	0	0	0	0	2	0	0	0
Corynebacterium pseudotuberculosis	0	3	0	0	0	0	0	1	0	0	0
Corynebacterium pyogenes	0	0	0	0	0	0	0	0	0	0	0
Corynebacterium ulcerans	0	1	0	0	0	0	0	1	0	0	0
Coxiella burnetti	0	0	0	0	0	0	0	0	0	0	0
Edwardsiella tarda	0	0	0	0	1	0	0	1	0	0	0
Ehrlichia sennetsu	0	0	0	0	0	0	0	0	0	0	0
Eikenella corrodens	0	0	0	1	0	0	0	0	0	0	0
Elizabethkingia meningoseptica	0	0	0	0	2	0	1	0	0	4	0
Enterobacter cloacae	1	0	0	0	1	1	3	4	10	4	0
Erysipelothrix rhusiopathiae	0	0	0	0	46	0	0	1	0	0	0
Escherichia coli	0	0	0	0	0	0	0	0	0	1	0
Flavobacterium meningosepticum	0	0	0	0	0	0	0	0	0	0	0
Fluoribacter bozemanae	0	0	0	0	0	0	0	0	0	0	0
Francisella tularensis	0	0	0	3	0	0	1	0	0	0	0

Table S7 Continued

Fusobacterium necrophorum	0	0	0	0	1	0	0	3	0	5	0
Gardnerella vaginalis	0	0	0	0	0	0	0	0	0	0	0
Haemophilus ducreyi	0	0	0	0	0	0	0	0	0	0	0
Haemophilus influenzae	0	0	1	0	0	0	0	0	0	0	0
Helicobacter pylori	0	0	0	0	2	0	0	1	0	0	0
Klebsiella oxytoca	0	0	0	0	3	1	8	3	0	2	0
Klebsiella pneumoniae	0	0	1	0	0	0	2	7	16	13	1
Legionella pneumophila	0	0	0	2	36	7	22	25	77	10	37
Leptospira interrogans	0	0	0	0	7	0	1	1	1	0	0
Listeria ivanovii	0	0	0	0	0	0	0	0	1	0	0
Listeria monocytogenes	0	0	0	0	1	0	0	0	0	0	0
Moraxella catarrhalis	0	1	47	4	2	6	0	6	4	5	4
Morganella morganii	0	0	0	0	1	1	1	0	3	1	0
Mycobacterium africanum	0	0	0	0	0	0	0	0	0	0	0
Mycobacterium avium	0	0	1	0	0	0	0	0	0	0	0
Mycobacterium bovis	0	0	0	0	0	0	0	0	0	0	0
Mycobacterium chelonae	0	0	0	0	0	0	0	0	0	0	0
Mycobacterium fortuitum	0	0	0	0	0	0	0	0	0	0	0
Mycobacterium kansasii	0	0	0	0	0	0	0	0	0	0	0
Mycobacterium leprae	0	0	0	0	0	0	0	0	0	0	0
Mycobacterium malmoense	0	0	0	0	0	0	0	0	0	0	0
Mycobacterium marinum	0	0	1	0	1	0	0	0	0	1	1
Mycobacterium microti	0	0	0	0	0	0	0	0	0	0	0
Mycobacterium paratuberculosis	0	0	0	0	0	0	0	0	0	0	0
Mycobacterium scrofulaceum	0	0	0	0	1	0	0	0	0	0	0
Mycobacterium simiae	0	0	0	0	0	0	0	0	0	13	0
Mycobacterium szulgai	0	0	1	0	0	0	0	0	0	1	0
Mycobacterium tuberculosis	0	0	0	0	0	0	0	0	0	0	0
Mycobacterium ulcerans	0	0	0	0	0	0	0	0	0	0	0

Table S7 Continued

Mycobacterium xenopi	0	0	0	0	0	0	0	0	0	0	0
Mycoplasma caviae	0	0	0	0	0	0	0	0	1	0	2
Mycoplasma hominis	0	0	0	0	2	0	0	0	1	0	0
Mycoplasma pneumoniae	0	0	0	0	0	1	0	0	0	0	0
Neisseria gonorrhoeae	0	0	0	0	0	0	0	0	0	0	0
Neisseria meningitidis	0	0	0	0	1	0	1	0	0	1	0
Nocardia asteroids	0	0	0	0	0	0	0	0	0	0	0
Nocardia braziliensis	0	0	0	0	0	0	0	0	0	0	0
Nocardia farcinica	0	0	0	0	0	0	0	0	0	0	0
Nocardia nova	0	0	0	0	0	0	0	1	0	0	0
Nocardia otitidiscaviarum	0	0	0	0	2	0	0	1	0	0	0
Pasteurella multocida	0	0	0	0	0	1	0	0	0	0	0
Peptostreptococcus anaerobius	0	0	0	0	1	0	0	0	0	0	0
Plesiomonas shigelloides	0	0	1	0	0	1	0	0	0	0	0
Proteus mirabilis	0	0	0	1	0	0	0	0	11	0	0
Proteus penneri	0	0	0	0	0	1	0	0	0	0	0
Proteus vulgaris	0	0	0	0	0	0	0	0	1	0	0
Providencia alcalifaciens	0	0	1	0	0	0	0	0	0	1	0
Providencia rettgeri	0	0	0	0	0	0	0	3	0	1	0
Pseudomonas aeruginosa	2	0	1434	2	5	14	12	47	8	43	0
Pseudomonas mallei	0	0	0	0	0	0	0	0	0	0	0
Pseudomonas pseudomallei	0	0	0	0	0	0	0	0	0	0	0
Rhodococcus equi	0	0	0	0	0	0	0	0	0	0	0
Rickettsia akari	0	0	0	0	7	0	0	0	0	0	0
Rickettsia canada	0	0	0	0	0	0	0	0	0	0	0
Rickettsia conorii	0	0	0	0	1	1	2	2	7	3	3
Rickettsia montana	0	0	0	0	0	0	0	0	0	0	0
Rickettsia mooseri	0	0	0	0	0	0	0	0	0	0	0
Rickettsia prowazekii	0	0	0	0	1	0	1	0	3	0	0

Table S7 continued

Rickettsia rickettsia	0	0	0	0	1	0	0	0	1	0	0
Rickettsia sennetsu	0	0	0	0	0	0	0	0	0	0	0
Rickettsia tsutsugamushi	0	0	0	0	0	0	0	0	0	0	0
Rickettsia typhi	0	0	0	0	0	0	0	0	0	0	0
Rochalimaea quintana	0	0	0	0	0	0	0	0	0	0	0
Salmonella arizonae	0	0	0	0	0	0	0	0	0	0	0
Salmonella enterica	0	0	0	3	2	0	1	1	0	2	1
Salmonella paratyphi	0	0	0	0	0	0	0	0	0	0	0
Salmonella typhi	0	0	0	0	0	0	0	0	0	0	0
Shigella boydii	0	0	0	0	0	0	0	0	0	0	0
Shigella dysenteriae	0	0	0	0	0	0	0	0	0	0	0
Shigella flexneri	0	0	0	0	2	0	0	0	0	1	0
Shigella sonnei	0	0	0	0	0	0	0	1	0	0	0
Staphylococcus aureus	0	0	0	0	0	0	0	0	1	0	0
Streptobacillus moniliformis	0	0	0	0	2	0	0	1	0	1	0
Streptococcus agalactiae	0	0	0	0	1	0	0	0	0	0	0
Streptococcus dysgalactiaeequisimilis	0	0	0	0	0	0	0	0	0	0	0
Streptococcus pneumoniae	0	1	14	0	1	2	0	3	0	1	0
Streptococcus pyogenes	0	0	0	0	0	0	0	0	0	1	0
Streptococcus suis	2	0	5	1	2	3	1	1	1	49	0
Treponema carateum	0	0	0	0	0	0	0	0	0	0	0
Treponema pallidum	0	0	0	0	0	0	0	0	0	0	0
Treponema pertenue	0	0	0	0	0	0	0	0	0	0	0
Ureaplasma parvum	0	0	0	0	0	0	0	0	0	0	0
Ureaplasma urealyticum	0	0	0	0	0	0	0	0	0	0	0
Vibrio cholerae	0	0	0	0	1	3	2	0	0	0	0
Vibrio parahaemolyticus	0	0	1	0	0	2	1	3	2	0	0
Yersinia enterocolitica	0	0	0	0	0	0	0	1	0	0	0

Table S7 continued

Yersinia pestis	0	0	0	0	0	0	0	0	0	0	0
Yersinia pseudotuberculosis	0	0	0	0	0	0	0	0	1	0	0
Total Number of Reads	**42370**	**113607**	**466260**	**171502**	**506157**	**266619**	**174893**	**230575**	**101137**	**76247**	**131952**

Table S8 Anions in the filtered water samples from 13 different sources, along with WHO and Nepalese guideline values where applicable. The water was analysed with two different methods; HACH Cuvette Test and Ion Chromatography. The analysis was performed in duplicate for HACH Cuvette Test and the number in parenthesis is the standard deviation.

	**HACH Cuvette Test**				**Ion Chromatography**						
**Sample Site ID**	**Fluoride (mg/L)**	**Ammonium (mg/L)**	**Nitrite (mg/L)**	**Nitrate (mg/L)**	**Fluoride (mg/L)**	**Chloride (mg/L)**	**Bromide (mg/L)**	**Nitrate (mg/L)**	**Phosphate (mg/L)**	**Nitrite (mg/L)**	**Sulphate (mg/L)**
1	<0.1	<0.019	<0.05	3.1501 (0.0726)	0.078	16.780	n.d.	2.083	0.061	0.062	9.670
2	<0.1	0.0492 (0.0015)	<0.05	<1	0.157	0.202	0.044	0.201	0.072	0.018	0.201
3	<0.1	<0.019	<0.05	<1	0.053	0.921	n.d.	0.513	0.033	0.014	0.584
4	<0.1	<0.019	<0.05	2.7228 (0.1560)	0.054	6.156	n.d.	1.979	0.082	0.021	7.730
5	0.378 (0.028)	<0.019	<0.05	1.4004 (0.0229)	0.369	2.048	0.012	0.036	17.162	0.015	0.366
6	0.22 (0.011)	0.2808 (0.015)	<0.05	<1	0.400	31.690	n.d.	0.155	0.240	0.092	62.580
7	<0.1	1.7791 (0.0316)	<0.05	2.1966 (0.0389)	0.091	38.488	0.713	0.877	0.054	0.136	20.246
8	<0.1	<0.019	<0.05	59.0741 (1.5943)	0.025	55.814	0.196	52.550	0.068	0.184	61.483
9	<0.1	<0.019	<0.05	1.5425 (0.1155)	0.035	0.995	n.d.	1.340	0.192	0.030	1.994
10	<0.1	0.03122 (0.0041)	<0.05	1.2926 (0.0125)	0.162	1.510	n.d.	0.710	0.144	0.025	2.609
11	<0.1	<0.019	0.0707 (0.002)	78.9677 (1.8065)	0.247	78.279	0.754	149.821	1.798	0.437	0.002
12	<0.1	0.0452 (0.0016)	<0.05	2.7410 (0.2453)	0.056	1.423	n.d.	1.801	0.055	0.010	5.098

Table S8 Continued

13	<0.1	<0.019	<0.05	2.01 (0.0879)	0.165	4.822	n.d.	1.462	0.296	0.035	6.826
WHO guidance value	1.5		3	50	1.5	250		50		3	
Nepalese guidance value		1.5		50				50			250

Table S9 Anions and cations used for charge balance error calculation. Total negative and positive charges are expressed as equivalent per liter.

Sample Site ID	Conductivity (µS/cm)	Alkalinity ($mgCaCO_3/L$)	Cl^- (mg/L)	pH	NO_3^- (mg/L)	SO_4^{2-} (mg/L)	Ca^{2+} (mg/L)	Mg^{2+} (mg/L)	K^+ (mg/L)	Na^+ mg/L)	Positive charges (eq./L)	Negative charges (eq./L)	Charge balance error (%)
1	216	41	16.780	8.2	3.099	9.670	33.338	2.916	1	2.434	0.002035	0.001544	13.724
2	8.1	4	0.202	6.5	1.000	0.201	0.493	0.100	1	0.492	0.000080	0.000106	-14.058
3	38.7	11	0.921	6.8	1.000	0.584	3.212	0.371	1	0.881	0.000255	0.000274	-3.659
4	268	98	6.156	7.8	2.833	7.730	40.195	4.378	1.2	4.232	0.002581	0.002339	4.926
5	1139	490	2.048	6.6	1.417	0.366	86.760	13.374	5.834	42.487	0.007427	0.009880	-14.170
6	805	340	31.690	6.7	1.000	62.580	98.192	14.267	15.994	32.686	0.007905	0.009007	-6.517
7	531	200	38.488	6.3	2.169	20.246	47.894	7.501	9.641	19.236	0.004091	0.005539	-15.039
8	662	130	55.814	6.2	57.947	61.483	57.293	12.639	15.990	33.802	0.005778	0.006387	-5.001
9	241	170	0.995	8.0	1.461	1.994	51.115	2.478	1	1.064	0.002827	0.003490	-10.505
10	250	126	1.510	7.9	1.284	2.609	48.745	2.916	1	1.236	0.002752	0.002635	2.160
11	670	91	78.279	6.7	77.690	0.002	59.175	12.923	27.984	56.250	0.007179	0.005279	15.247
12	217	104	1.423	7.5	2.568	5.098	35.122	6.952	1	0.902	0.002390	0.002266	2.658
13	62.2	14	4.822	6.2	1.948	6.826	4.168	1.117	1	4.661	0.000528	0.000589	-5.470

Table S10 Metals in the filtered water samples from 13 different sources, along with WHO and Nepalese guideline values where applicable.

	**Sample Site ID**													**Guidance Value**	
**Cations**	**1**	**2**	**3**	**4**	**5**	**6**	**7**	**8**	**9**	**10**	**11**	**12**	**13**	**WHO Guidance Value**	**Nepal Guidance Value**
**Total Ca (mg/L)**	33.338	0.493	3.212	40.195	86.760	98.192	47.894	57.293	51.115	48.745	59.175	35.122	4.168	300	200
**Total Mg (mg/L)**	2.916	<0.1	0.371	4.378	13.374	14.267	7.501	12.639	2.478	2.916	12.923	6.952	1.117		
**Total Na (mg/L)**	2.434	0.492	0.881	4.232	42.487	32.686	19.236	33.802	1.064	1.236	56.250	0.902	4.661		
**Total K (mg/L)**	<1	<1	<1	1.2	5.834	15.994	9.641	15.990	<1	<1	27.984	<1	<1		
**Total Ba (mg/L)**	0.026	0.002	0.004	0.036	0.095	0.186	0.118	0.121	0.0375	0.0331	0.1236	0.0487	0.0102	0.7	
**Total Fe (mg/L)**	0.004	<0.001	<0.001	0.003	0.6656	0.1973	1.4810	0.1617	0.0426	0.0565	0.0036	0.0083	0.0022		0.3
**Total Mn (mg/L)**	0.002	<0.001	<0.001	0.001	0.5281	0.1192	0.6568	0.0501	0.0086	0.0062	<0.001	0.0013	0.0025	0.1	0.2
**Total Al (mg/L)**	0.1379	<0.005	<0.005	0.214	<0.005	0.0055	0.0062	0.0071	0.0116	0.0096	<0.005	0.0078	0.0106	0.2	0.2
**Total Zn (mg/L)**	0.004	<0.002	<0.002	0.027	0.0034	0.0234	0.0027	<0.002	<0.002	0.0035	0.0021	0.1374	0.0099		3

Table S10 Continued

**Total Pb (mg/L)**	<0.001	<0.001	<0.001	<0.001	<0.001	<0.001	<0.001	<0.001	<0.001	<0.001	<0.001	<0.001	<0.001	0.01	0.01
**Total Cu (mg/L)**	<0.001	0.003	<0.001	<0.001	<0.001	0.0056	<0.001	<0.001	<0.001	<0.001	0.0021	0.0019	<0.001	2	1
**Total As (mg/L)**	<0.005	<0.005	<0.005	<0.005	<0.005	<0.005	<0.005	<0.005	<0.005	<0.005	<0.005	<0.005	<0.005	0.01	0.05
**Total Cd (mg/L)**	<0.0001	<0.0001	<0.0001	<0.0001	<0.0001	<0.0001	<0.0001	<0.0001	<0.0001	<0.0001	<0.0001	<0.0001	<0.0001	0.003	0.003
**Total Sb (mg/L)**	<0.005	<0.005	<0.005	<0.005	<0.005	<0.005	<0.005	<0.005	<0.005	<0.005	<0.005	<0.005	<0.005	0.02	
**Total Si (mg/L)**	4.3374	0.1797	0.5792	5.7317	35.2107	14.3344	22.1596	10.2059	4.1522	4.1550	14.0731	3.6717	5.3907		
**Total Ni (mg/L)**	<0.001	<0.001	<0.001	<0.001	0.0015	0.0017	0.0016	0.0012	<0.001	<0.001	<0.001	<0.001	<0.001	0.07	
**Total Cr (mg/L)**	<0.001	<0.001	<0.001	<0.001	<0.001	<0.001	<0.001	0.001	<0.001	<0.001	<0.001	<0.001	<0.001	0.05	0.05
**Total Sr (mg/L)**	0.044	<0.001	0.003	0.064	0.2271	0.3163	0.1870	0.1952	0.0402	0.0392	0.1290	0.0847	0.0247		

Table S11 List of water borne diseases endemic in the Kathmandu Valley and their causative agents.

**Waterborne Disease**	**Causative Agents**	**References**
Diarrhoea	*Campylobacter*, *Clostridium difficile, Escherichia coli, Salmonella, Shigella, Vibrio cholera*	[7-9]
Cholera	*Vibrio cholerae*	
Typhoid	*Salmonella enterica, Salmonella typhi, Salmonella paratyphi*	
Dysentry	*Campylobacter*, *Escherichia coli, Shigella, Salmonella*	
Gastroenteritis	*Campylobacter jejuni, Escherichia coli, Salmonella, Shigella, Clostridium difficile, Staphylococcus aureus*	

Table S12 Real-time qPCR primers for different genetic markers

Target Organisms	Primer	Sequence (5'>>>3')	Annealing Temperature (T_a)	Amplicon size	Reference
Vibrio cholera	EpsM -F	GAATTATTGGCTCCTGTGCAGG	57 °C	248	[10]
	EpsM -R	ATCGCTTGGCGCATCACTGCCC			
Total Coliform	Eco1457F	CATTGACGTTACCCGCAGAAGAAGC	60 °C	190	[11]
	Eco1652R	CTCTACGAGACTCAAGCTTGC			
Total *E.coli*	rodA -F	GCAAACCACCTTTGGTCG	60 °C	194	[12]
	rodA -R	CTGTGGGTGTGGATTGACAT			
Human *E.coli*	Hu 8 -F	ACAGTCAGCGAGATTCTTC	60 °C	177	[13]
	Hu 8 -R	GAACGTCAGCACCACCAA			
	Hu 100 –F	ACGGTTATCAGCTCACGTCG	60 °C	98	(Robson and Davenport, in preparation)
	Hu 100 -R	TCGCCCCTCGAAAAGCATTA			
Total Bacteria (16S rRNA); qPCR	1055 F	ATGGCTGTCGTCAGCT	60 °C	337	[14]
	1392 R	ACGGGCGGTGTGTAC			
16S rRNA amplicon sequencing; MinION	27F	AGAGTTTGATCMTGGCTCAG	55 °C	1500	Oxford Nanopore Technologies, UK
	1492R	CGGTTACCTTGTTACGACTT			
16S rRNA amplicon sequencing; Illumina	515 F	GTGCCAGCMGCCGCGGTAA	55 °C	291	[15]
	806 R	TAATCTWTGGGVHCATCAGG			

References

1 APHA. Standard Methods for the Examination of Water and Wastewater. 18th edition. American Public Health Association. (1992).

2 WHO. Guidelines for drinking-water quality: fourth edition incorporating the first addendum. Geneva: World Health Organization (2017).

3 DWSS. National Drinking Water Quality Standards. Kathmandu: Department of Water Supply and Sewerage (2005).

4 Shrestha, S. M., Rijal, K. & Pokhrel, M. R. Assessment of Heavy Metals in Deep Groundwater Resources of the Kathmandu Valley, Nepal. *Journal of Environmental Protection* **7**, 516 (2016).

5 Zymo Research. ZymoBIOMICS™ Microbial Community Standard, Catalog No. D6300 (2019).

6 HSE. The Approved List of biological agents. Third edition. Merseyside: Health and Safety Executive. (2013).

7 Leclerc, H., Schwartzbrod, L. & Dei-Cas, E. Microbial agents associated with waterborne diseases. *Critical reviews in microbiology* **28**, 371-409 (2002).

8 Karkey, A. *et al.* The ecological dynamics of fecal contamination and Salmonella Typhi and Salmonella Paratyphi A in municipal Kathmandu drinking water. *PLoS neglected tropical diseases* **10**, e0004346 (2016).

9 http://www.waterwise.co.za/site/water/diseases/waterborne.html (accessed 2019).

10 Kong, R. Y. C., Lee, S. K. Y., Law, T. W. F., Law, S. H. W. & Wu, R. S. S. Rapid detection of six types of bacterial pathogens in marine waters by multiplex PCR. *Water research* **36**, 2802-2812 (2002).

11 Bartosch, S., Fite, A., Macfarlane, G. T. & McMurdo, M. E. T. Characterization of bacterial communities in feces from healthy elderly volunteers and hospitalized elderly patients by using real-time PCR and effects of antibiotic treatment on the fecal microbiota. *Applied and environmental microbiology* **70**, 3575-3581 (2004).

12 Chandrashekhar, K. M. *et al.* Limit of detection of genomic DNA by conventional PCR for estimating the load of Staphylococcus aureus and Escherichia coli associated with bovine mastitis. *Folia microbiologica* **60**, 465-472 (2015).

13 Gomi, R., Matsuda, T., Matsui, Y. & Yoneda, M. Fecal source tracking in water by next-generation sequencing technologies using host-specific Escherichia coli genetic markers. *Environmental science & technology* **48**, 9616-9623 (2014).

14 Harms, G. *et al.* Real-time PCR quantification of nitrifying bacteria in a municipal wastewater treatment plant. *Environmental science & technology* **37**, 343-351 (2003).

15 Kozich, J. J., Westcott, S. L., Baxter, N. T., Highlander, S. K. & Schloss, P. D. Development of a dual-index sequencing strategy and curation pipeline for analyzing amplicon sequence data on the MiSeq Illumina sequencing platform. *Appl. Environ. Microbiol.* **79**, 5112-5120 (2013).
